# Transcriptomic profiling of granuloma in patients with cardiac sarcoidosis

**DOI:** 10.7150/thno.109211

**Published:** 2025-04-28

**Authors:** Praveen Gajawada, Stefan Günther, Andreas Rolf, Jamal Nabhanizadeh, Rajkumar Savai, Yeong-Hoon Choi, Manfred Richter

**Affiliations:** 1 Department of Cardiac Surgery, Kerckhoff Heart Center, Benekestr. 2-8, Bad Nauheim 61231, Germany.; 2 Campus Kerckhoff, Justus-Liebig-University Giessen, 61231 Bad Nauheim, Germany.; 3 Department of Cardiology, Kerckhoff Heart and Lung Center, Benekestrasse 2-8, Bad Nauheim 61231, Germany.; 4 Bioinformatics and Deep Sequencing Platform, Max Planck Institute for Heart and Lung Research, Bad Nauheim 61231, Germany.; 5 Cardio- Pulmonary Institute, Bad Nauheim 61231, Germany.; 6 German Center for Cardiovascular Research (DZHK), Partner Site RhineMain, Frankfurt/Main, Germany.; 7 Max Planck Institute for Heart and Lung Research, Member of the German Center for Lung Research (DZL), Member of the Cardio-Pulmonary Institute (CPI), Bad Nauheim, 61231, Germany.; 8 Institute for Lung Health (ILH), Justus Liebig University, 35392, Giessen, Germany.

**Keywords:** colony stimulating factor 1 receptor, interleukin-7 receptor, interleukin-4 receptor, chitinase-3-like protein 1, chitotriosidase-1, chemoattraction, m2 macrophage, immune cells, human leukocyte antigen, inflammation

## Abstract

Cardiac sarcoidosis (CS) is an inflammatory condition characterized by the accumulation and clustering of immune cells, primarily macrophages, leading to granuloma formation. Despite its clinical significance, CS remains relatively understudied, particularly concerning the molecular mechanisms driving fibrosis and disease progression. To explore potential therapeutic targets, we aimed to characterize the transcriptomic landscape of CS granulomas.

**Methods:** We performed RNA sequencing, immunostaining, and Western blot analysis on granulomatous tissue from explanted CS hearts. We used myocardial tissue from patients with aortic stenosis, preserved ejection fraction, and normal myocardium as a reference group. Gene ontology analysis was conducted, and upstream pathway analysis was performed to determine key modulators driving gene expression within the granulomas.

**Results:** RNA sequencing revealed differential gene expression patterns in granulomatous tissue, highlighting a distinct set of up- and down-regulated genes. Specifically, we observed significant expression of human leukocyte antigens, chitinase-3-like protein 1 (CHI3L1), chitotriosidase-1 (CHIT1), and several immunoglobulin genes within macrophages. Gene ontology analysis identified the activation of immune response pathways, signaling cascades, and cytokine secretion mechanisms. Upstream pathway analysis identified CSF1R as a primary regulator of gene expression, with IL7R also playing a role. The predominance of M2-associated transcripts suggests that granulomas exhibit an anti-inflammatory and tissue remodeling phenotype, whereas the presence of M1-associated transcripts indicates an early inflammatory response that may transition to an M2 phenotype over time.

**Conclusions:** Our findings provide new insights into the immune landscape of CS granulomas and highlight the role of macrophage polarization in granuloma expansion and fibrosis development. The identification of CSF1R as a key upstream regulator, together with the prominence of CHI3L1 and CHIT1, highlights potential targets for therapeutic intervention. These findings support the notion that an M1 to M2 transition may drive fibrotic remodeling, ultimately contributing to heart failure.

## Introduction

Sarcoidosis was first described in the late 19^th^ century. However, its specific involvement in the heart, known as cardiac sarcoidosis (CS), gained clearer recognition in the 20^th^ century as diagnostic methods improved. Initially, the understanding of CS was limited due to its often asymptomatic nature and the challenges of diagnosing granulomas within the cardiac tissue 1. The histological characteristics of sarcoidosis granulomas encompass the presence of well-organized layers of immune cells, arranged in concentric patterns, with a notable central core consisting of aggregates of macrophages and multi-nucleated giant cells. The mechanism underlying the formation and function of these giant cells in granulomatous structures, especially in humans, is not fully elucidated 2. At the molecular level, the pathogenesis of CS is marked by the activation of antigen-presenting cells (APCs), including macrophages and dendritic cells, in the heart tissue. These cells process and present antigens to T lymphocytes, initiating an immune response. The interaction between APCs and T cells is crucial for the formation of granulomas, and various cell signaling molecules, including cytokines and chemokines mediate it. Tumor necrosis factor-alpha (TNF-α) and interleukin-1 (IL-1) are key pro-inflammatory cytokines that have been implicated in the inflammatory processes of CS 3, 4. Previously, we described macrophages to be attracted primarily through chemoattraction rather than proliferation and accumulate in areas of remodeling cardiomyocytes with elevated levels of oncostatin M (OSM) and the chemokines Reg3A and Reg3γ 5. Under chronic conditions, cardiomyocytes degenerate through OSM, releasing macrophages and, in turn, massive fibrosis develops 6, 7. T-helper 1 (Th1) cells secrete a specific set of cytokines, including interferon-gamma (IFN-γ), which further stimulates the immune response against the myocardium, contributing to the formation of granulomas 8. IFN-γ also increases the expression of major histocompatibility complex (MHC) molecules on APCs, enhancing antigen presentation 9. Another crucial aspect of the molecular mechanisms in cardiac sarcoidosis is the dysregulation of fibroblast function in connection with the production and remodeling of the extracellular matrix (ECM) remodeling. Although the cause of fibrosis in CS remains largely unknown, growing evidence indicates that macrophages, especially those polarized toward the M2 phenotype, may contribute to promoting the transition to fibrosis in later stages of disease progression 10. We intended to extend our previous results 5 by performing transcriptomic analysis of granuloma from patients with cardiac sarcoidosis. We sought to identify key regulatory genes in granulomas that might reveal dysregulated pathways and molecular signatures associated with CS and elucidate the complex interactions between different immune cell subsets, cytokines, and signaling pathways involved in granuloma development and expansion in CS.

## Materials and Methods

### CS patients and experimental approach

We analyzed 4 explanted hearts from patients with cardiac sarcoidosis and left ventricular ejection fraction lower than 25% who underwent heart transplantation. The clinical parameters of the analyzed patients have been described in detail previously elsewhere 5. Using a stereomicroscope, the granulomas were excised precisely from frozen heart tissue samples and subjected to whole transcriptome RNA sequencing. Excising the entirety of granulomatous structures for RNA sequencing offers a comprehensive snapshot of gene expression across all cell types within the tissue, providing insights into the complex interplay and functional dynamics between various cell populations, thus providing deeper insights into the molecular mechanisms driving the disease. Cardiac tissue samples from patients with aortic stenosis, preserved ejection fractions, and no inflammatory signs were used as reference (EF > 50%; CTRL, n = 3).

### RNA sequencing and gene ontology analysis

For RNA-sequencing, total RNA was isolated from adult human hearts using Trizol reagent. RNA and library preparation integrity were verified using the BioAnalyzer 2100 (Agilent) or LabChip Gx Touch 24 (Perkin Elmer). Approximately, 1 ng of total RNA was used as input for SMART-Seq® v4 Ultra® Low Input RNA Kit (Takara Clontech) for cDNA pre-amplification. Obtained full-length cDNA was checked on LabChip and fragmented by Ultrasonication by E220 machine (Covaris). The final library preparation was performed by Low Input Library Prep Kit v2 (Takara Clontech). Sequencing was performed on the NextSeq500 instrument (Illumina) using v2 chemistry with a 1x75bp single-end setup. The resulting raw reads were assessed for quality, adapter content, and duplication rates with FastQC 28. Reaper version 13-100 was employed to trim reads after a quality drop below a mean of Q20 in a window of 10 nucleotides (Davis *et al.*, Kraken: A set of tools for quality control and analysis of high-throughput sequence data). Only the reads between 30 and 150 nucleotides were cleared for further analyses. Trimmed and filtered reads were aligned versus the mouse genome version mm10 (GRCm38) using STAR 2.4.0a with the parameter “--outFilterMismatchNoverLmax 0.1” to increase the maximum ratio of mismatches to mapped length to 10% (Dobin *et al.*, STAR: ultrafast universal RNA-seq aligner). The number of reads aligning to genes was counted with the feature Counts 1.4.5-p1 tool from the Subread package 29. Only reads mapping at least partially inside exons were admitted and aggregated per gene. Reads overlapping multiple genes or aligning to multiple regions were excluded. The Ensemble annotation was enriched with UniProt data (release 06.06.2014) based on Ensembl gene identifiers (Activities at the Universal Protein Resource (UniProt)). DEGs were submitted to gene set enrichment analyses with the KEGG Orthology-Based Annotation System (KOBAS) 30. Two separate tests were performed per contrast using only either up- or down-regulated genes for analysis. The results were combined keeping only gene sets that showed significant overrepresentation at False Discovery Rate (FDR) <0.05 in only one input list (i.e. that were either enriched for up- or down-regulated genes, but not both). The Top10 gene sets considering enrichment FDR were selected per the direction of regulation.

Upstream pathway analysis was analyzed using Ingenuity Pathway Analysis (QIAGEN Inc., https://www.qiagenbioinformatics.com/products/ingenuity-pathway-analysis).

### Immunofluorescence staining and fluorescence microscopy

Fixation, staining, and microscopy were reported previously 31. The following antibodies were used for immunofluorescence staining: Versican Recombinant Rabbit Monoclonal Antibody (Catalog# MA5-34654, Invitrogen), Fibronectin (Catalog# F14420-050, BD), CD3 (Catalog# M7254, Dako), CD4 (Catalog# F0766, Dako), CD8 (Catalog# M7103, Dako), CD68 (Catalog#M0718, Dako), Mast cell tryptase (Catalog# ABIN2475503, Antikörper-Online), CD11c (Catalog# ABIN6939818, Antikörper-Online), HLA-DQA1 (Catalog# MA532833, Invitrogen), HLA-DPB1 (Catalog# PA582411, Invitrogen), HLA-DQB1 (Catalog# PA578003, Invitrogen), IL7Rα (Catalog# ab180521, Abcam), Chitinase 3-like-1 (Catalog# AF2599, Biotechne), Chitotriosidase 1 (Catalog# 214321-AP, Proteintech), IL4Rα (Catalog# ab131058, Abcam), IL13Rα1 (Catalog# ab63436, Abcam), Phospho-Jak3 (Tyr980/981) (Catalog# 5031, Cell Signaling), Phospho-STAT6 (Tyr641) (Catalog# 9361, Cell Signaling), Acetyl-Histone H3 (Lys9) (Catalog# 9671, Cell Signaling), TGFß (Catalog# 3711, Cell Signaling), Alexa Fluor™ 633 Phalloidin (F-actin) (Catalog#A22284, Invitrogen), and DAPI (4',6-Diamidino-2-Phenylindole, Dilactate) (Catalog# D3571, Invitrogen).

### Protein extraction and Western blot analysis

Protein extraction from the frozen heart tissues was done by sonicating in protein extraction buffer (0.1 M Tris-HCl pH 8.8, 0.01 M EDTA, 0.04 M DTT, 10 % SDS, pH 8.0) supplemented with protease inhibitors (0.5 mg/ml Benzamidine, 2 μg/ml Aprotinin, 2 μg/ml Leupeptin, 2 mM PMSF, 1 mM Sodium Vanadate, 20 mM Sodium Fluoride). Proteins were separated using SDS-PAGE on Gradient NuPAGE 4-12% Bis-Tris gels (Invitrogen) followed by blotting onto nitrocellulose membranes (Invitrogen). After blocking with the milk powder, the membranes were incubated with specific primary antibodies, and imaging was done VersaDoc (Bio-Rad). The antibodies used for western blot were: IL7Rα (Catalog#ab180521, Abcam), Phospho-IL7Rα (Tyr449) (Catalog#PA5-38619, ThermoFisher Scientific), Phospho-Jak2 (Tyr1008) (D4A8) (Catalog#8082, Cell Signaling), Phospho-STAT5 (Tyr694) (D47E7) XP^®^ (Catalog#4322, Cell Signaling), Phospho-Jak3 (Tyr980/981) (Catalog#5031, Cell Signaling), Phospho-STAT6 (Tyr641) (Catalog#9361, Cell Signaling), GAPDH (D16H11) XP^®^ (Catalog#5174, Cell Signaling), Hexokinase I (C35C4) (Catalog#2024, Cell Signaling).

## Results

### Transcriptomic analysis of granuloma from hearts of cardiac sarcoidosis patients

**Figure 1:**
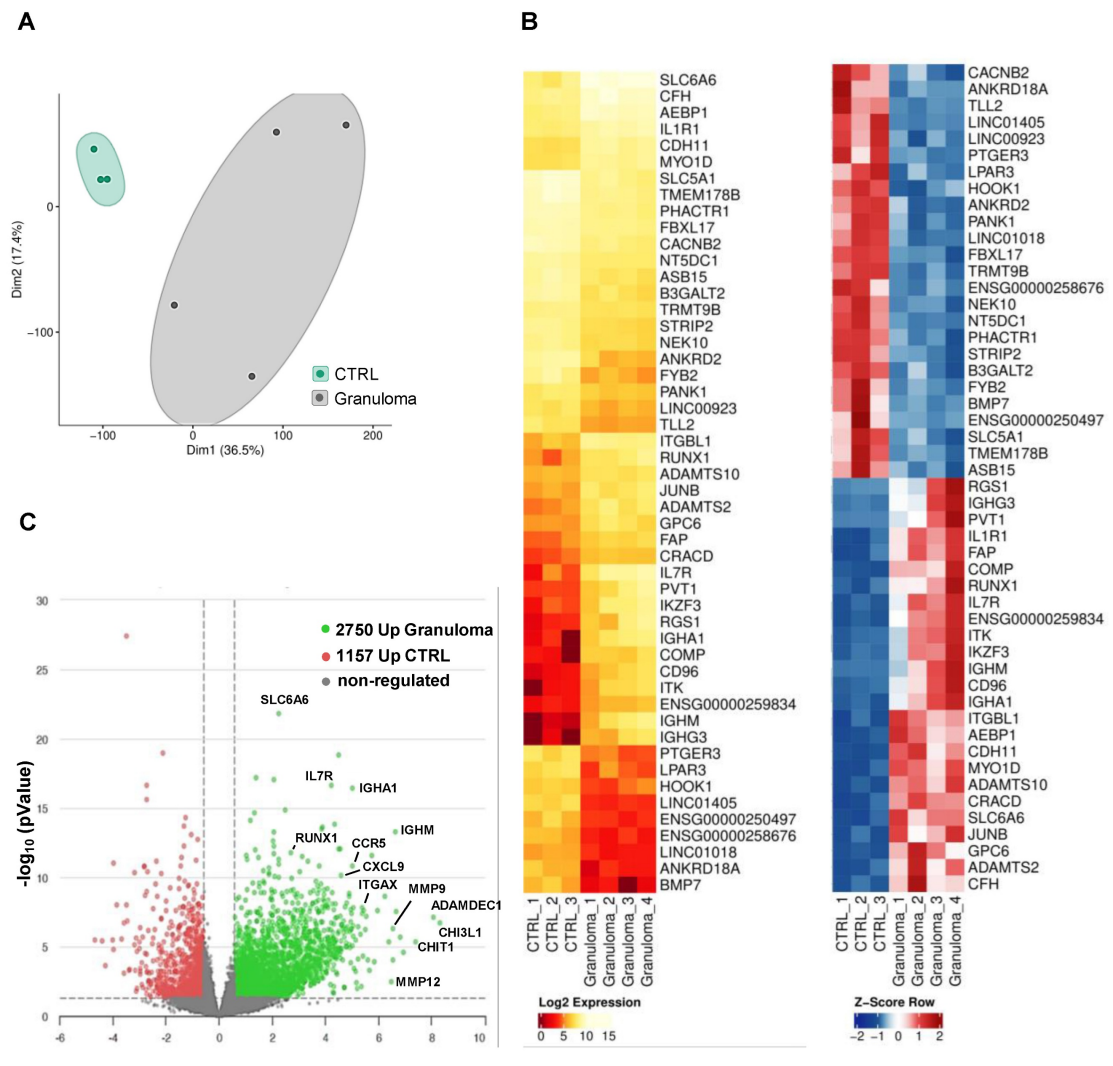
** Transcriptomic profiling of RNA from cardiac granuloma and control myocardium.** (**A**) Principal component analysis plot of CTRL (control, n=3) and granuloma (n=4). (**B**) Heat map of the top 25 differentially expressed genes (DEG) with FDR<0.05, log2 fold change <>+-0.585, and mean count >5 plotted as log2 expression (left) and Z-Score (right). (**C**) Volcano plot depicting fold changes of DEG in CTRL and granuloma with P value ≤ 0.05 and log10 fold change <>+-0.585.

The whole transcriptome RNA-seq analysis revealed many differentially expressed genes (DEG) in granulomas. In a two-dimensional principal component analysis, the CTRL group exhibited a closely clustered pattern, while the granuloma group displayed a more dispersed clustering, suggesting differences in transcriptomes (Figure 1A). A list of the top 50 DEGs for granuloma versus CTRL is shown as a hierarchical heatmap in Figure 1B. Compared to the reference group, the granuloma exhibited higher expression levels in 2,750 genes and lower levels in 1,157 genes. (Figure 1C).

**Figure 2:**
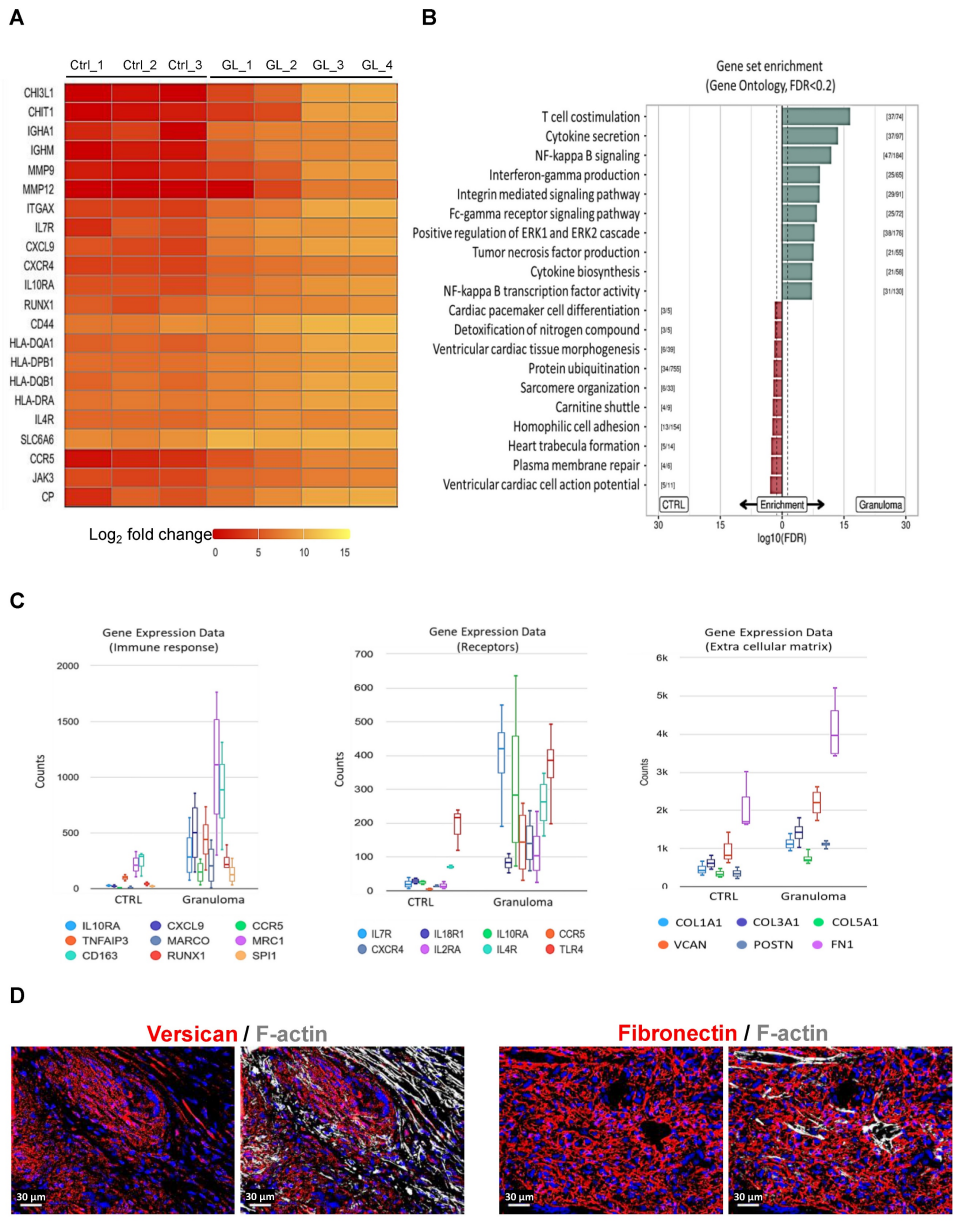
** Gene expression pattern and gene set enrichment analysis of granuloma.** (**A**) Heat map of selective significantly differentially expressed genes. Log2 fold values are represented with colors ranging from red (low expression), orange (moderate expression), and yellow (high expression. (**B**) Directional gene set overrepresentation analysis using KOBAS found pathways that were enriched for DEGs in CTRL (red) or granuloma (green) conditions at an FDR < 0.2. The dashed line represents an FDR of 0.05, while the values in brackets denote the number of DEGs versus total genes in the respective pathway. The top 10 pathways per direction were selected. (**C**) Box plot graphs showing the relative counts derived from RNA sequencing data, depicting the expression patterns of immune response genes, receptors, and extracellular matrix genes. The counts in granuloma for all the genes are significantly higher than in CTRL, adjusted P value ≤ 0.05. (**D**) Enhanced extracellular matrix deposition in granuloma visible by the expression of Versican and Fibronectin. The nuclei are stained blue with DAPI.

Interestingly, Chitotriosidase 1 (CHIT1) and chitinase-3-like protein 1 (CHI3L1) are significantly upregulated in cardiac granuloma (Figure 2A), which are also expressed in granuloma of other organs. Similar to other forms of sarcoidosis, the expression of RUNX1, MMP9, MMP12, CCR5, ITGAX, CD44, and human leukocyte antigens (HLA) is also seen in the granuloma of CS (Figure 2A). Moreover, other selectively up-regulated genes are shown as heat maps in Figure 2A. Gene ontology analysis using KOBAS shows the top 10 pathways that are enriched for DEGs up-regulated in CTRL and granuloma (Figure 2B). Selective genes upregulated in granuloma were linked to various biological processes including immune response (IL10RA, CXCL9, CCR5, TNFAIP3, MARCO, MRC1, and CD163), surface receptors (IL7Rα, IL4R, IL18R1, IL10RA, CCR5, CXCR4, IL2RA, and TLR4), and ECM deposition (COL1A1, COL3A1, COL5A1, VCAN, POSTN, and FN1) (Figure 2C). Immunofluorescence staining showed a massive presence of versican (VCAN) and fibronectin (FN1) in the granulomatous environment (Figure 2D).

### Composition and abundance of immune cells in cardiac granuloma

**Figure 3:**
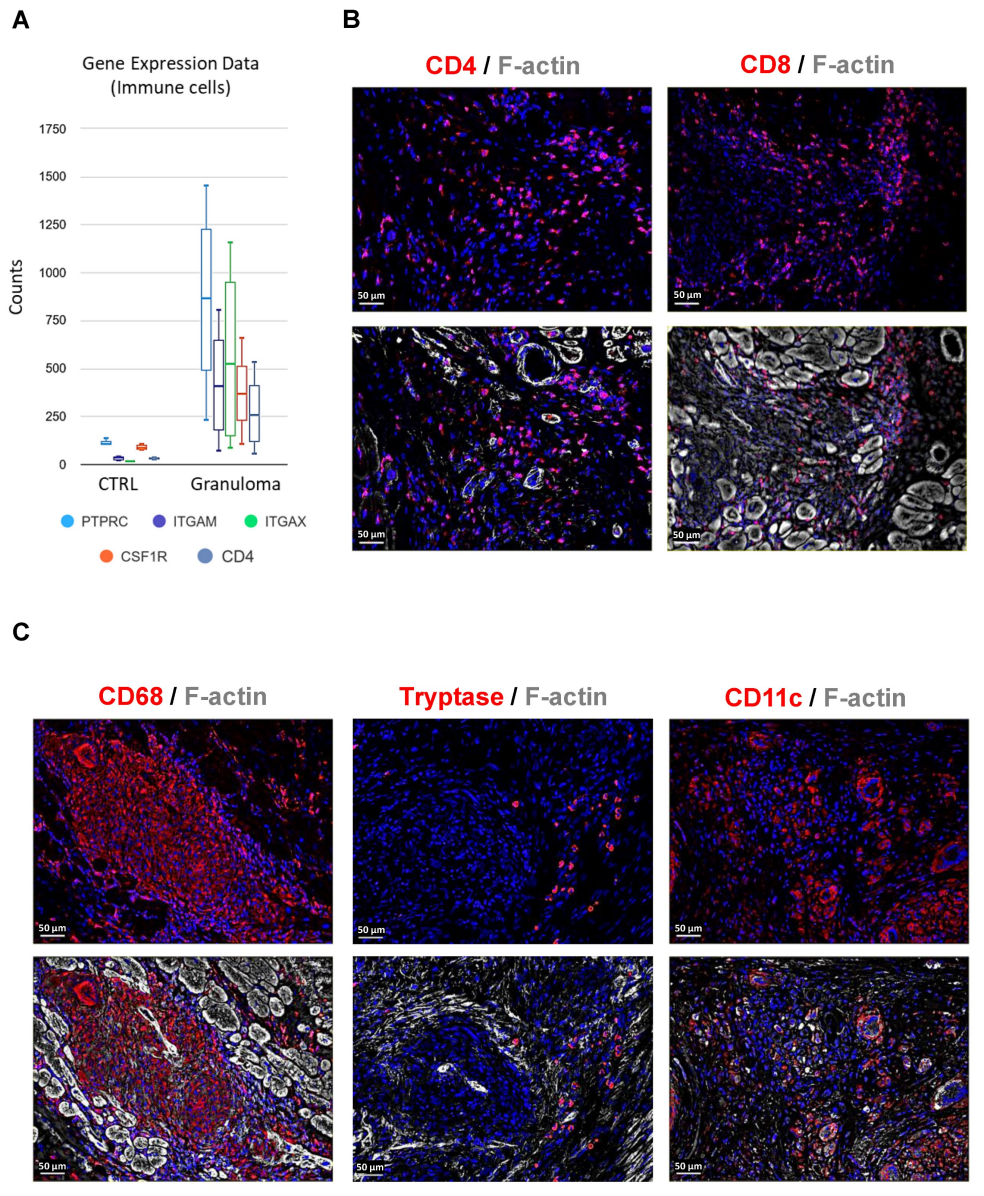
**Composition of immune cells in granuloma.** (**A**) Graph illustrating the relative counts derived from RNA sequencing data, showcasing the expression profiles of immune cell markers, significantly expressed in granuloma with an adjusted P value ≤ 0.05. (**B**) Immunofluorescence staining of granuloma reveals CD4 and CD8 positive T lymphocytes are sparsely distributed in the granuloma. (**C**) Macrophages (CD68^+^) are overwhelmingly abundant within the granuloma, while mast cells (Tryptase^+^) are scarcely observed, and dendritic cells (CD11C^+^) are moderately present. The nuclei are stained blue with Dapi.

RNA sequencing of granuloma shows the expression of immune cell marker genes such as PTPRC (CD45), ITGAM (CD11b), ITGAX (CD11c), CSF1R (CD115), and CD4 (Figure 3A). The observations in end-stage cardiac sarcoidosis regarding the composition of granulomas reflect a distinctive immune profile. CD4^+^ and CD8^+^ T lymphocytes are sparsely located within the granuloma (Figure 3B). Interestingly, mast cells were identified within the granuloma setting, yet their sparse presence at the core of the granuloma underscores a distinct immune microenvironment that deviates from conventional granulomatous inflammation (Figure 3C). The presence of moderately abundant dendritic cells suggests their involvement in antigen presentation and immune regulation within the granulomatous lesions (Figure 3C). Overall, the granulomas in end-stage cardiac sarcoidosis exhibit a skewed immune cell composition characterized by moderately present T lymphocytes, overwhelming macrophages, sparse mast cells, and moderate dendritic cell presence, highlighting a distinct immunopathological profile.

### Macrophages in granuloma express various human leukocyte antigens (HLAs) and immunoglobulin-related genes

**Figure 4:**
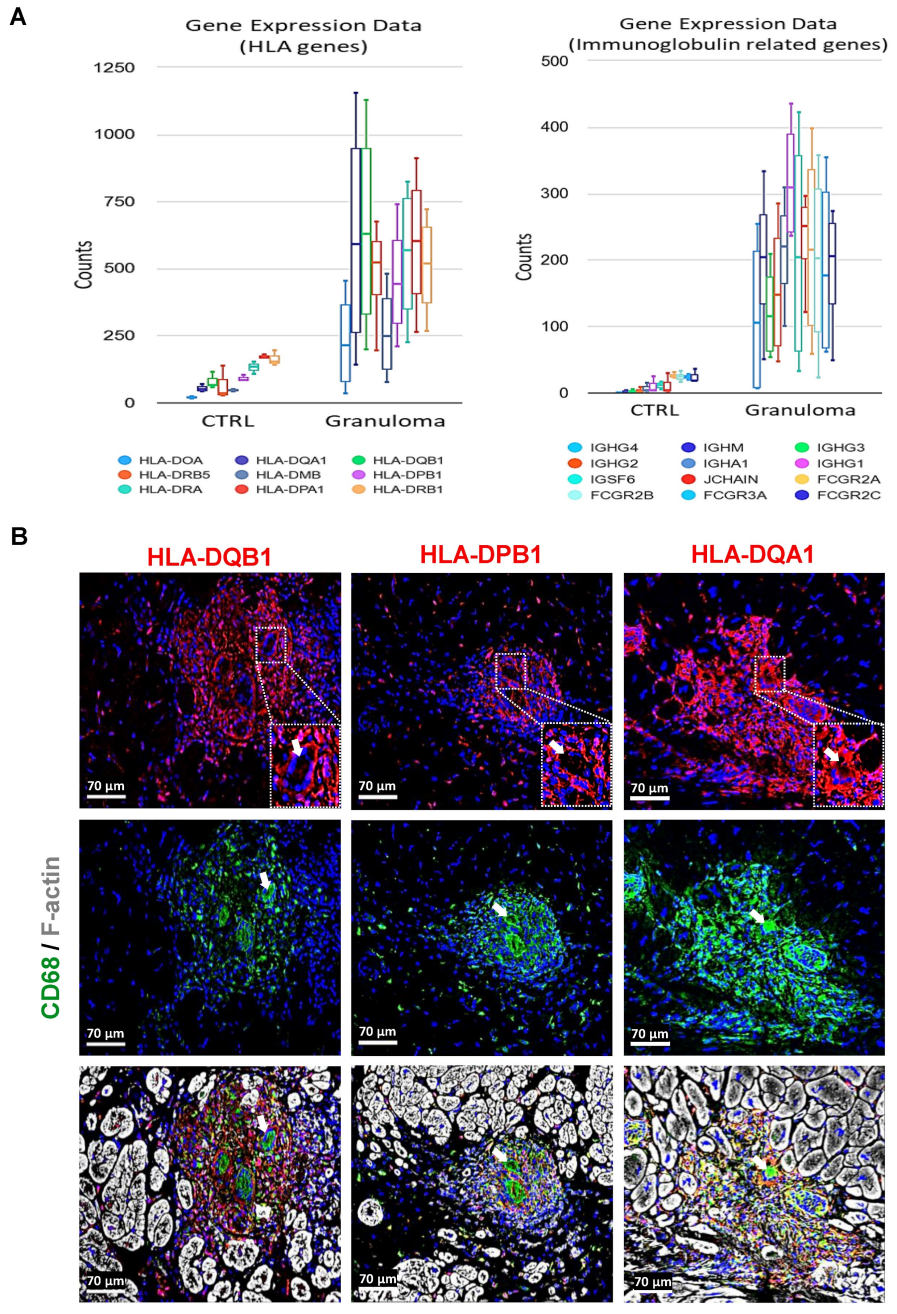
** Human leukocyte antigen and immunoglobulin-related genes are strongly expressed in granuloma.** (**A**) Charts depicting the results obtained from RNA sequencing data, highlighting the enhanced expression of HLAs and immunoglobulin-related genes. The counts for all the genes are significantly higher in granuloma, adjusted P value ≤ 0.05. (**B**) Immunofluorescence images show a strong expression of HLA-DQB1, HLA-DPB1, and HLA-DQA1 in the macrophages (CD68^+^), but not in the giant cells (enlarged area with white arrows). The nuclei are stained blue with Dapi.

The observed upregulation of several HLA genes and immunoglobulin-related genes suggests a heightened immune response within the granulomatous microenvironment (Figure 4A). The absence of B lymphocytes despite the upregulation of immunoglobulin-related genes raises questions regarding the contribution of humoral immunity in cardiac sarcoidosis. Local factors within the cardiac microenvironment may dictate the immune cell composition and response patterns, leading to unique immunopathological features distinct from other forms of sarcoidosis. Immunofluorescence staining further elucidates the localization of HLA expression within the granuloma. The elevated expression of HLA-DQB1, HLA-DPB1, and HLA-DQA1 specifically in macrophages, rather than giant cells (indicated with white arrows), underscores the dynamic interplay between different immune cell populations within the granulomatous lesions (Figure 4B). Macrophages, known for their phagocytic and antigen-presenting capabilities, appear to be the primary drivers of HLA-mediated immune responses in this context. These findings align with the literature indicating the central role of macrophages in the pathogenesis of sarcoidosis, particularly in the context of granuloma formation.

### CSF1R is a key upstream regulator driving M2 macrophage dominance in granulomas

**Figure 5:**
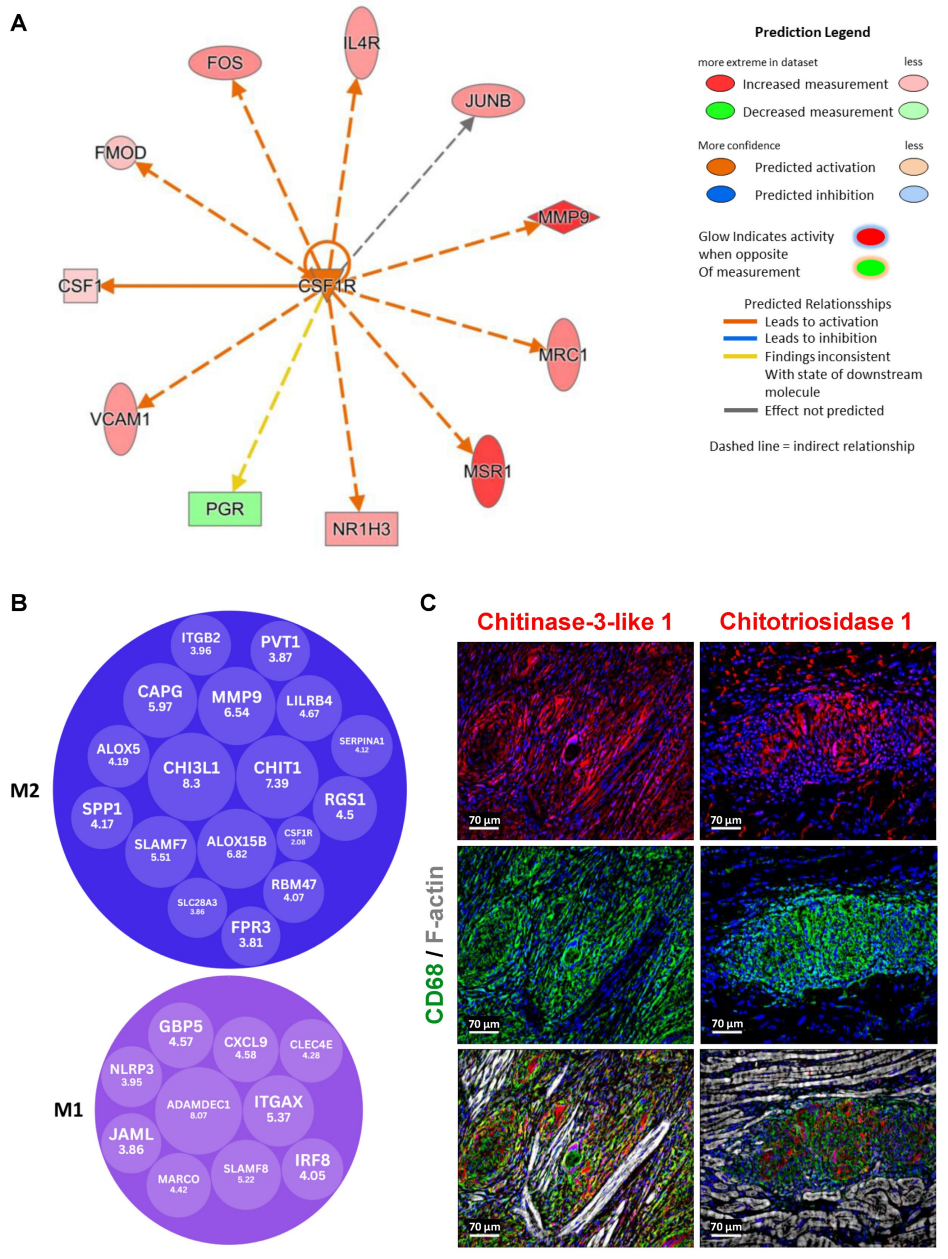
** Granulomas exhibit predominant expression of M2 genes, including Chi3l1 and Chit1, highlighting their abundance compared to M1 genes.** (**A**) Upstream pathway analysis identified CSF1R as a significant upstream regulator in granuloma versus control DEGs (P-value < 0.05, fold change cutoff between -0.5 and 0.5). These predictions were based on statistical evaluation of DEGs known to be regulated by each cytokine or transcription factor, calculated using the overlap P-value. The upstream regulators are visualized through relational networks, providing insights into their connections and functional pathways. (**B**) The evaluation of DEGs related to M1/M2 polarization was performed through supervised annotation of the top 50 highly expressed genes identified in granuloma versus control DEGs. Among these top 50 genes, 10 are recognized as M1 polarization markers, while 17 are identified as M2 polarization markers, based on published reference research (CSF1R is not in the top 50 genes). The results indicate a predominance of M2 genes, both in terms of number and higher expression levels (log2 fold change) in granuloma compared to the control. (**C**) The immunofluorescence images demonstrate marked expression of Chi3l1 and Chit1 in macrophages within granulomas, validating transcriptomic analysis.

Upstream pathway analysis using Ingenuity Pathway Analysis (IPA, Qiagen) revealed that CSF1R is a significant upstream regulator in granulomas compared to control samples (Figure 5A). This analysis was based on the statistical evaluation of DEGs known to be regulated by various cytokines and transcription factors, using the overlap P-value method. The upstream regulators were visualized using both relational and mechanistic networks, which provided valuable insights into their connections and functional pathways. Notably, the mechanistic networks identified clusters of interconnected regulators that could collectively trigger the observed gene expression changes. These findings align with current literature, which implicates the CSF1/CSF1R axis in directing macrophage differentiation and influencing tissue homeostasis, suggesting that CSF1R-mediated signaling is pivotal in shaping the granulomatous response 11. Our upstream pathway analysis of CSF1R identified IL4R as a regulated gene, corroborating the literature linking interleukin-4 receptor alpha (IL4RA) and interleukin-13 receptor alpha 1 (IL13RA1) signaling to granuloma. In our study, we observed an increased expression of IL4RA and IL13RA1 in granuloma macrophages, as well as increased levels of phosphorylated Janus kinase 3 (P-JAK3) and phosphorylated STAT6 (P-STAT6) (Suppl. Figure 1). Importantly, although previous studies have confirmed the involvement of these receptors in granulomas, our data reveal a novel aspect: the spatial expression pattern shows that activated JAK3/STAT6 signaling largely overlaps with IL4R/IL13R expression but is conspicuously excluded from giant cells. Furthermore, elevated levels of Acetyl Histone H3 (Lys9) (Acetyl-H3K9) and TGFß were observed in the granuloma (Suppl. Figure 3).

A focused evaluation of DEGs related to macrophage polarization was performed via supervised annotation of the highly expressed genes in granulomas. Of these, 10 genes were recognized as M1 polarization markers, while 17 were identified as M2 markers, based on established literature (Figure 5B). Importantly, M2 genes were not only more but also exhibited higher log₂ fold changes compared to M1 markers. The transcriptomic analyses were further validated by immunofluorescence staining showing marked expression of Chi3l1 and Chit1 in macrophages within granulomas (Figure 5C).

### IL7RA is predominantly expressed and activated in macrophages

**Figure 6:**
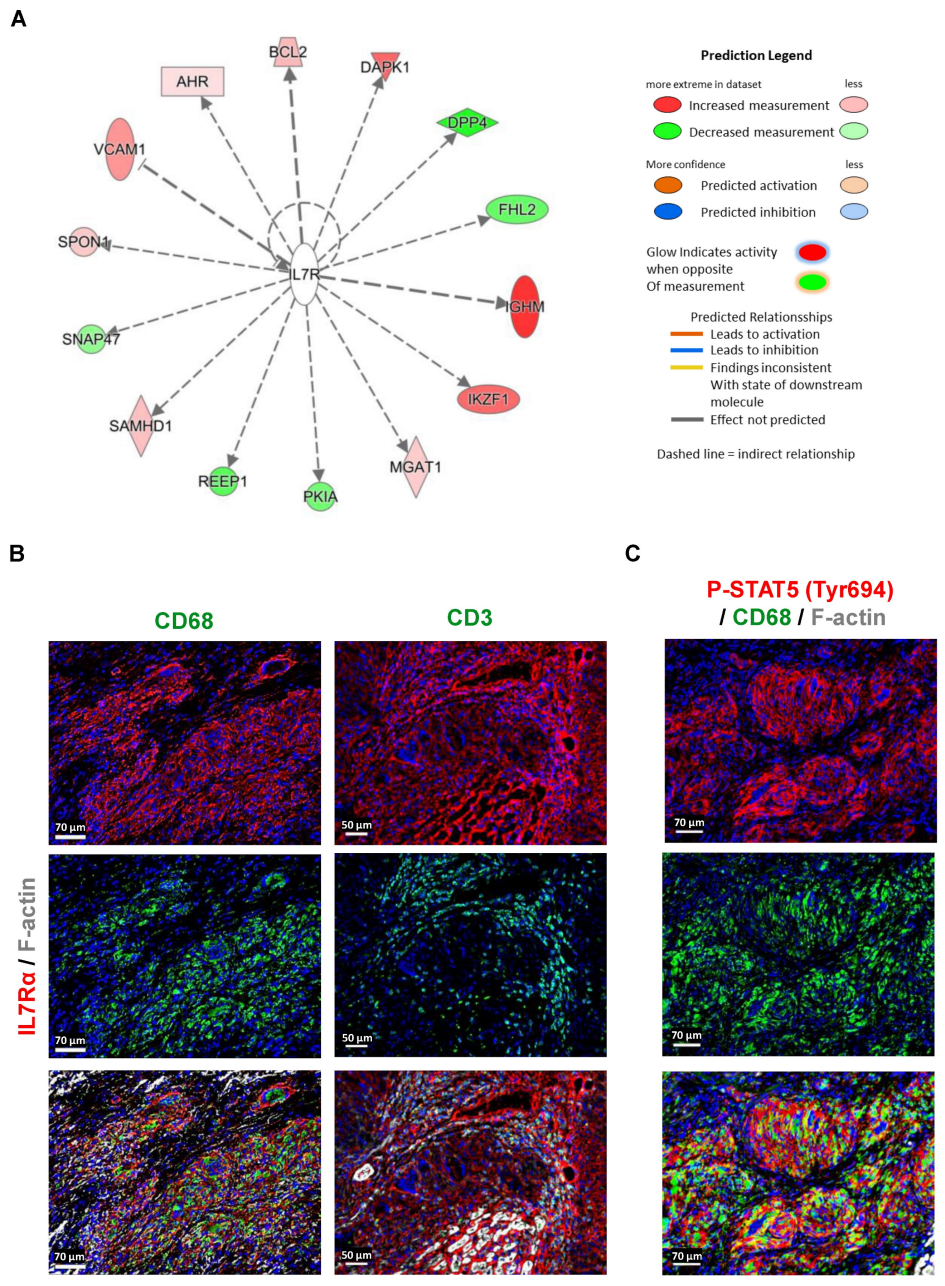
**Abundant expression and activation of IL7Rα cascade in macrophages of granuloma.** (**A**) Pathway analysis identified IL7R as an upstream regulator in granulomas compared to control DEGs (P-value < 0.05, fold change cutoff between -0.5 and 0.5). These findings were derived from the statistical evaluation of DEGs regulated by specific cytokines or transcription factors, calculated using the overlap P-value. Visualization of upstream regulators was carried out through relational networks, offering insights into their interactions and functional pathways. (**B**) Immunofluorescence staining of IL7Rα in macrophages (CD68^+^) and T lymphocytes (CD3^+^). (**C**) Immunofluorescence images show the expression of P-STAT5 (Tyr694) in macrophages (CD68^+^).

Pathway analysis also identified interleukin-7 receptor alpha (IL7Rα) as an upstream regulator in granulomas compared to control samples. This analysis, based on the statistical evaluation of DEGs regulated by specific cytokines or transcription factors, revealed IL7R's pivotal role in orchestrating gene expression changes. Visualization through relational and mechanistic networks provided insights into the functional pathways and interactions of IL7R, highlighting clusters of interconnected upstream regulators that may drive granuloma formation and progression. The observation of abundant expression of IL7Rα in macrophages within the granulomatous lesions, as revealed by RNA-seq analysis and immunofluorescence imaging, holds considerable significance (Figure 1C, 2A, 2C, 6A, and 6B). While IL7Rα expression has been previously associated with T lymphocytes 12, this study marks the first demonstration of its prominent presence in macrophages within the context of cardiac sarcoidosis. The high expression of IL7Rα in macrophages, coupled with the comparatively lower presence of T lymphocytes, hints at a distinctive role for IL7Rα signaling in macrophage-driven immune responses within the granuloma. Furthermore, the western blot analysis revealed increased levels of phosphorylated IL7Rα (P-IL7Rα), total IL7Rα, phosphorylated Janus kinase2 (P-JAK2), and phosphorylated signal transducer and activator of transcription (P-STAT5), suggesting a potential activation of the JAK2/STAT5 pathway by IL7Rα signaling in macrophages (Figure 6B, 6C, and Suppl. Figure 2). Collectively, these results imply a functional involvement of IL7Rα in orchestrating macrophage responses within the granuloma.

## Discussion

The transcriptomic analysis of granulomas from the hearts of CS patients provides valuable insights into the transcriptional and immunological mechanisms underlying the disease. This study significantly contributes to the limited literature on the transcriptomic landscape of CS, which has been sparse until now. To the best of our knowledge, only a single instance of transcriptomic analysis of CS is known that has mainly employed single-nucleus RNA sequencing (snRNA-seq) and CD68-based spatial transcriptomics 13. In contrast, this study utilizes whole-tissue RNA sequencing to capture the entirety of granulomatous structures' transcriptomic signatures.

The composition and abundance of immune cells within cardiac granulomas highlight a distinctive immune profile in end-stage CS. Macrophages are overwhelmingly abundant, suggesting a predominant role of macrophage-driven processes in the pathogenesis. The presence of mast cells along with dendritic cells, T lymphocytes, and macrophages is a novel finding as not much is known of their presence in sarcoidosis 14. It prompts further investigation into the interactions between mast cells and other immune cells within the granuloma, as well as their influence on disease progression and outcomes. Our earlier finding, which revealed that the primary cellular constituents of cardiac granulomas are mature macrophages expressing CD68, CD168, and CD206, and with fewer CD3/CD4/CD8 positive T-cells 5, was supported by the expression of various macrophage-associated genes such as RUNX1, MARCO, CD163, and SPI1 (transcription factor PU.1). This holds particular significance given that IL7Rα expression was predominantly observed in macrophages, despite T-cells and B cells being traditionally considered the primary immune cells expressing IL7Rα 15. Although we previously observed similar distributions of this receptor in patients with end-stage myocarditis, the general morphologic features of immune cells between these two heart diseases are very different 5, 16. Immunostaining and western blot analysis further corroborated the high IL7Rα protein expression in macrophages. Activation of the receptor occurs through binding with its ligand interleukin-7 (IL7), initiating crosslinking with the common γ-chain and resulting in specific phosphorylation events such as Tyr499 on IL7Rα. Two major pathways, Jak/STAT, and PI3K/Akt, are known to be activated by IL7 in T lymphocytes 17. We have observed higher levels of P-Jak2 and P-STAT5 in macrophages (Suppl. Figure 2) and our previous findings have shown that the PI3K/Akt pathway is highly activated in granuloma macrophages and might contribute to sustaining the high activity and viability of granulomata by suppressing cell death proteins 5. Taken together, the high expression and activation of IL7Rα, PI3K/Akt, and Jak2/STAT5, we infer that the IL7 signaling pathway is activated in macrophages within the granuloma of patients with heart failure.

The most striking observation from our analysis is the significant involvement of CSF1R, as identified through upstream pathway analysis. The CSF1R pathway plays a crucial role in macrophage biology, particularly in promoting their survival and differentiation toward an M2 phenotype. Literature has established that CSF1R signaling is essential for maintaining tissue-resident macrophages, and it is closely associated with the anti-inflammatory and reparative functions characteristic of M2 macrophages. For example, Leblond *et al.* (2015) demonstrated that inhibition of CSF1R signaling depleted M2 macrophages in cardiac tissue, resulting in impaired wound healing and enhanced fibrotic remodeling following myocardial infarction 18. Research evaluating the therapeutic benefits of modulating CSF1R activity emphasizes that the receptor not only maintains macrophage viability but also promotes their differentiation into an M2 phenotype, which plays a crucial role in tissue repair and immune regulation 19.

In the context of CS granulomas, our findings of enhanced CSF1R expression suggest that robust CSF1R signaling may create a microenvironment conducive to the accumulation of M2 macrophages. This is further supported by the concurrent high expression of IL4R, a receptor that drives macrophage polarization toward the M2 state through its interaction with IL4, a cytokine known to induce anti-inflammatory functions and pro-fibrotic mediator secretion 33. The synergistic activation of CSF1R and IL4R pathways in granuloma-associated macrophages likely contributes to the persistence and expansion of these lesions while promoting tissue remodeling and fibrosis, ultimately impacting cardiac function. This dual regulatory mechanism underscores a potential target for future therapeutic interventions aimed at modulating macrophage responses in cardiac sarcoidosis.

Additionally, we observed an enhanced expression of IL13Rα1, Jak3, and STAT6 in macrophages within CS granulomas and the increased phosphorylation of Jak3 and STAT6 are indicative of an activation of anti-inflammatory IL4/IL13 signaling pathways. This finding highlights the role of the Jak3/STAT6 signaling cascade in macrophages within granulomas, distinct from giant cells. STAT6 activation is an important downstream mediator of IL4/IL13 responses in these cells, supporting its role in driving alternative (M2) macrophage activation in sarcoidosis 34. This finding further reinforces our previous postulation that the anti-inflammatory state of granuloma macrophages could be influenced by increased expression of the interleukin-1 receptor antagonist (IL-1ra) 5. The spatial organization of IL4Rα and IL13Rα1 expression around giant cells suggests a potential paracrine signaling mechanism between macrophages and giant cells, further underscoring the complex cellular interactions and anti-inflammatory signaling within granulomatous lesions. Exposure to IL4 and IL13 can trigger the induction of M2 macrophages. These cytokines facilitate the transformation of macrophages into an anti-inflammatory state, characterized by the secretion of molecules like IL10 and pro-fibrotic by transforming growth factor beta (TGF-β) secretion 20. Moreover, elevated levels of Acetyl-H3K9 may indicate transcriptional activation of genes involved in macrophage polarization. This modification is often associated with the expression of anti-inflammatory and pro-fibrotic genes. Furthermore, Acetyl-H3K9 could be linked to the activation of pathways such as IL4/IL13 signaling 32. Together, these findings not only extend our understanding of the molecular mechanisms driving cardiac sarcoidosis but also highlight potential targets, specifically the CSF1R and IL4R/Jak3/STAT6 pathways, for therapeutic intervention aimed at modulating macrophage responses and mitigating tissue damage.

Among the other differentially expressed genes, notable upregulation of CHIT1 and CHI3L1 is observed in cardiac granulomas. Chitotriosidase 1 (CHIT1) is a chitinase that has been found to be a useful biomarker in sarcoidosis, with its activity correlating with disease activity, severity, and multiorgan dissemination 21, 22. CHIT1 has been reported to be significantly upregulated in patients with sarcoidosis, and its expression is strongly associated with the activation state of macrophages 22. Chitinase 3-like-1 (CHI3L1), another glycoprotein, is also implicated in the pathogenesis of various human diseases characterized by inflammation and remodeling, including sarcoidosis 23, 24. CHI3L1, also known as YKL‑40, has been implicated in tissue remodeling and the expansion of fibrosis through its effects on extracellular matrix dynamics and fibroblast activity. Elevated levels of CHI3L1 are associated with increased secretion of fibrogenic cytokines such as TGF‑β, which stimulates collagen deposition and promotes fibrotic remodeling in various chronic inflammatory conditions 25. In the context of cardiac sarcoidosis, the upregulation of CHI3L1 within granulomas may help create a pro-fibrotic microenvironment that supports the persistence of M2 macrophages and drives fibrotic expansion, ultimately contributing to adverse cardiac remodeling and dysfunction. Based on our current findings and the literature, we propose CHI3L1 as a candidate biomarker, while noting that further longitudinal studies and treatment-response analyses are necessary to definitively establish its role in disease progression and therapeutic outcomes.

Enhanced expression of collagens, periostin, and fibronectin have been extensively described in various forms of sarcoidosis. However, there is no study linking versican, a critical extracellular matrix regulator of immunity and inflammation, to any type of sarcoidosis. Versican interacts with inflammatory cells either indirectly via hyaluronan or directly via receptors such as CD44, P-selectin glycoprotein ligand-1 (PSGL-1), and toll-like receptors (TLRs) present on the surface of immune and non-immune cells and are also up-regulated in RNA-seq data. These interactions activate signaling pathways that promote the synthesis and secretion of inflammatory cytokines 26. Given the role of versican in inflammation and immune regulation, it's plausible that this matrix regulator is involved in the pathogenesis of sarcoidosis. The concurrent expression of Acetyl-H3K9, Chi3l1, CSF1R, IL4R, TGFß, and Jak3/Stat6 in granulomas of CS further reinforces macrophages towards M2 polarization, thereby leading to anti-inflammatory states and fibrosis. These molecular players create a microenvironment conducive to granuloma persistence, extracellular matrix deposition, and fibrotic expansion, ultimately contributing to cardiac dysfunction in sarcoidosis. This highlights potential therapeutic targets, such as modulating CSF1R or IL4R/Jak3-Stat6 signaling, to mitigate fibrosis and its impact on cardiac function.

Our gene ontology analysis showed the enrichment of pathways such as NF-kappa B signaling, integrin-mediated signaling, Fc-gamma receptor signaling, and the Erk1/2 cascade (Figure 2B). We have shown earlier that the NF-kappa B cascade is activated in only a few cells at the periphery of granuloma, whereas the Erk1/2 cascade was massively activated 5. Conversely, pathways related to sarcomere organization and cardiac cell action potential indicate an intact myocardium in the reference hearts. The enhanced expression of immune-related genes like CCR5, CXCR4, TLR4, IL2RA, CD163, MRC1, SPI1, and Marco indicate that immune cells are highly active entities in the granuloma. The upregulation of several HLA genes and immunoglobulin-related genes in macrophages within CS granulomas underscores the heightened immune response within these lesions. Conventionally, HLA molecules play a crucial role in antigen presentation, facilitating the activation of T lymphocytes, particularly CD4^+^ helper T cells. The absence of B lymphocytes despite the upregulation of immunoglobulin genes raises questions regarding the role of humoral immunity in CS. Interestingly, Fc gamma receptor (FcγR) genes expression indicates a highly phagocytic environment in the granuloma of CS, which is similar to what is known in pulmonary sarcoidosis 27. Immunofluorescence staining confirms the localization of HLA-DQB1, HLA-DPB1, and HLA-DQA1 expression specifically in macrophages within granulomas, emphasizing their role as primary drivers of HLA-mediated immune responses. As far as we know, previous research has only discussed the expression of HLA-DQB1 13. However, our study presents new evidence for the expression of HLA-DPB1 and HLA-DQA1 in macrophages within CS granulomas. Interestingly, all the HLA genes are not expressed by the giant cells, but only by surrounding macrophages.

The results from this study align with and extend previous literature on the molecular and cellular mechanisms underlying CS. This study offers a comprehensive analysis of the transcriptomic landscape of CS granulomas using whole-tissue RNA sequencing. The observed immune cell composition and expression of key genes in CS granulomas corroborate observations from other forms of sarcoidosis, indicating common pathways driving granuloma formation across different organ systems. The dysregulation of gene expression, distinctive immune cell composition, and spatial activation differences of specific signaling pathways within granulomas highlight the complex nature of CS pathogenesis. These findings contribute to our understanding of CS pathophysiology and may pave the way for the development of targeted therapeutic interventions aimed at modulating immune responses and tissue remodeling processes within granulomatous lesions.

## Conclusions

This study provides a comprehensive analysis of the transcriptomic landscape analysis of cardiac sarcoidosis (CS) granulomas using whole-tissue RNA sequencing combined with antibody-based assays. The concurrent expression of Acetyl-H3K9, Chi3l1, CSF1R, IL4R, TGFß, and Jak3/Stat6 in granulomas of cardiac sarcoidosis promotes the polarization of macrophages toward the M2 phenotype, facilitating anti-inflammatory conditions and driving fibrotic processes. The increased expression of CSF1R, IL7R, and IL4R in macrophages, together with the activation of their signaling cascades, suggests a critical role for these receptors in the pathogenesis of cardiac sarcoidosis. While inflammation certainly plays a crucial role in the development of a granuloma, inflammatory processes may play a lesser role when the granuloma is fully formed. Based on this work and our earlier study, we therefore assume that patients with cardiac sarcoidosis should be treated differently in the early and late stages.

## Supplementary Material

Supplementary figures.
