# Supplementary material for: Transcriptomic profiling of granuloma in patients with cardiac sarcoidosis
Source: Theranostics. 2025 Apr 28;15(13):6044–57. doi: 10.7150/thno.109211 (PMC12159750; doi:10.7150/thno.109211)
Supplement: Supplementary file 1 — Supplementary figures. [file thnov15p6044s1.pdf]

Supplementary Figures:

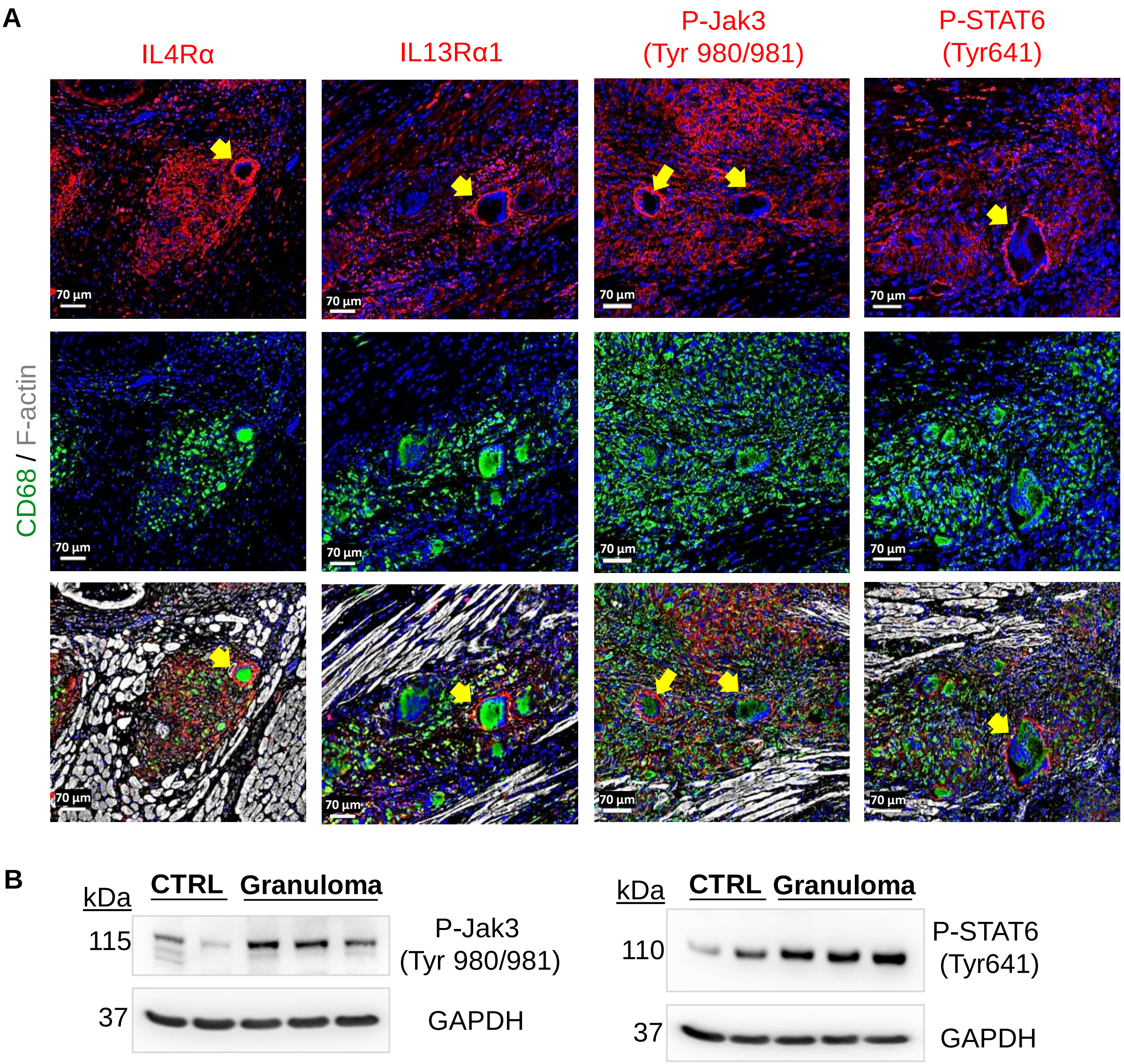

Suppl. Figure 1: **Activation of JAK3/STAT6 signaling and the spatial organization of IL4Rα and IL13Rα1 expression within granulomas.** (**A**) Enhanced expression of IL4Rα, IL13Rα1, P-JAK3, and P-STAT6 in macrophages ($CD68^+$) within the granulomatous microenvironment. A marked increase in the expression of these proteins surrounding giant cells (yellow arrows). (**B**) Western blot analysis shows increased expression of P-JAK3 (Tyr980/981) and P-STAT6 (Tyr641) in the granuloma of CS hearts.

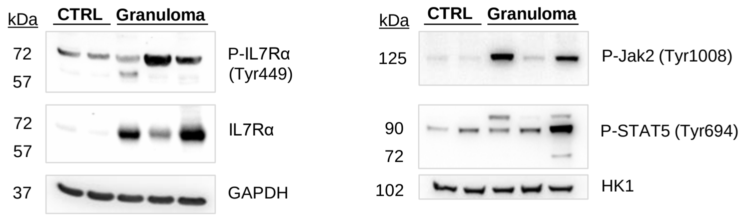

Suppl. Figure 2: **Abundant expression and activation of IL7Rα cascade in macrophages of granuloma.** Western blot analysis of P-IL7Rα (Tyr449), total IL7Rα, P-JAK2 (Tyr1008), and P-STAT5 (Tyr694) in granuloma of CS hearts. The nuclei are stained blue with Dapi.

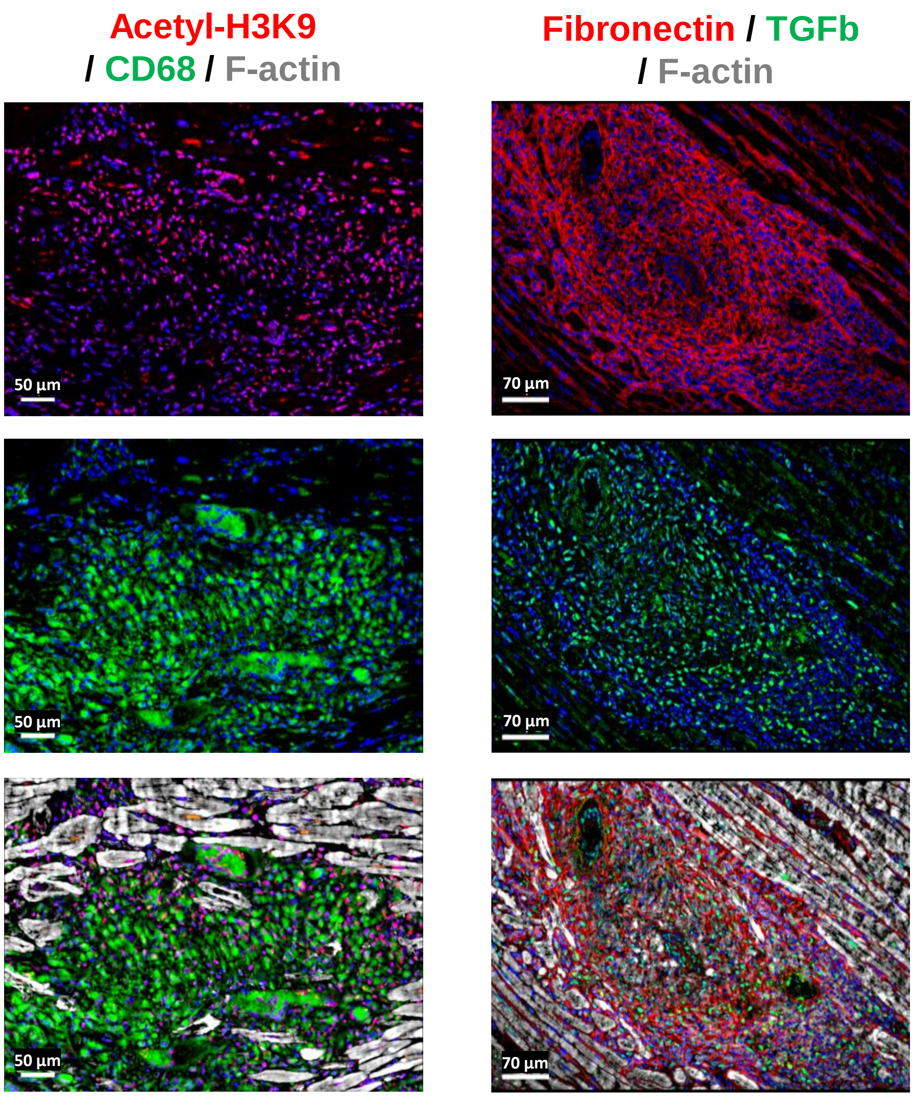

Suppl. Figure 3: **Expression of Acetyl-H3K9 and TGFß further reinforces fibrosis in granuloma.** Enhanced expression of Acetyl-H3K9 in macrophages ($CD68^+$) and TGFß in granuloma of CS hearts.
